# Sprinting with bilateral transtibial running-specific prostheses versus biological limbs – are they comparable? Comments on Beck *et al*. (2022)

**DOI:** 10.1098/rsos.230086

**Published:** 2023-09-06

**Authors:** M. J. Connick, E. M. Beckman, S. M. Tweedy

**Affiliations:** ^1^ School of Exercise and Nutrition Sciences, Queensland University of Technology, Queensland, Australia; ^2^ School of Human Movement and Nutrition Sciences, The University of Queensland, Queensland, Australia

**Keywords:** running, prosthetic, bilateral amputee, track, 400 m, para athletics

## Abstract

Since publication of this paper (Royal Society open science, 2022. **9**(1): p. 211799), the authors have published a correction clarifying that the paper presents a case study that ‘… did not meet the definition for research with regard to human subjects'. The data are incorrectly referred to as experimental because the study has no experimental control. Furthermore, the paper has been presented previously but the version presented here selectively omits several analyses, posing a significant risk of bias. Of the prosthetic-related disadvantages identified by the authors, the most substantive was a 40% increase in time to 20 m (59.5 s.d. below the mean for NA sprinters). However the analysis was incomplete: acceleration modelling for NA sprinters continued up to 98% of maximum velocity, while Fastest BA was truncated at approximately 80%. We extrapolated the model, revealing the duration of maximum acceleration for Fastest BA is approximately 100% longer than NA sprinters. Important differences in Fastest BA contact lengths (0.10–0.15 m) were also identified. We posit that together, these large and important differences in sprint biomechanics and their likely physiological consequences suggest that running with and without prosthetics are so different that, although running times may be similar, the precautionary principle should apply and, in the interests of athletic competition integrity, runners with and without prosthetics should continue to compete separately.

## 1. General comments

Several weaknesses in the study presented in ‘Sprinting with prosthetic versus biological legs: insight from experimental data’ significantly reduce the strength and generalizability of the findings. Firstly, we note that the authors have recently published a correction [1]. In it they clarified that, when the University of Colorado Boulder Institutional Review Board assessed their application (Protocol 18-0456), they determined it was a case study and ‘…did not meet the definition for research with regard to human subjects' [1].

Specifically, the authors present a descriptive case study, a design with no experimental control. Therefore the data collected should not be described as ‘experimental’ in the title or anywhere else in the article because it artificially inflates the strength of the evidence presented. Given the Boulder Institutional Review Board did not regard this case description as research, findings should be interpreted cautiously.

We also note that the authors had presented the data acquired from Protocol 18-0456 previously, in their capacity as expert witnesses in the Court of Arbitration for Sport (CAS) hearing ‘Blake Leeper v. International Association of Athletics Federations' [2]. In the CAS hearing the authors made it clear that the data were collected to determine whether the Ottobock 1 E90 prosthetics used by fastest BA provided him with a competitive advantage in the 400 m sprint. They included analyses which, they argued, showed that Fastest BA's prosthetics resulted in a 1.81 second disadvantage over 400 m and that, if Fastest BA had biological legs, he would be capable of running the 100 m in a world record (WR) 9.5 s and the 400 m in a WR 42.57 s (Para. 373). The CAS panel rejected this conclusion and found that, on the balance of probabilities, The Fastest BA's prosthetics actually conferred an advantage (not a disadvantage), at least partly because they were disproportionately long, a factor that the authors neglected to assess and which has been shown to be critically important [3].

In the RSOS article the authors alter the aims of the study so that they are not limited to Fastest BA only. They also omit the results of several key analyses, including those presented above [4]. These changes are important because, compared with *a priori* aims and analyses, the *post-hoc*, retro-fitted aims and selective presentation of analyses introduce a significant risk of bias, further weakening the strength of the evidence supporting the conclusions.

## 2. Comments on the 20 m acceleration analysis

The authors indicate that, of the disadvantages in 400 m sprinting caused by The Fastest BA's prosthetics, the most significant was his slow initial acceleration. Specifically, the authors report that in a maximum acceleration test, The Fastest BA was slower to reach 20 m than both elite (40%) and sub-elite (32%) non-amputee (NA) sprinters [2,4] and that, of the 1.81 s overall disadvantage which they attributed to The Fastest BA's prosthetics, 1.41 s (78%) was accrued because of his relatively poor acceleration out of the blocks [2]. However, as we explain below, the authors analysis was fundamentally flawed.

**Figure 1.**
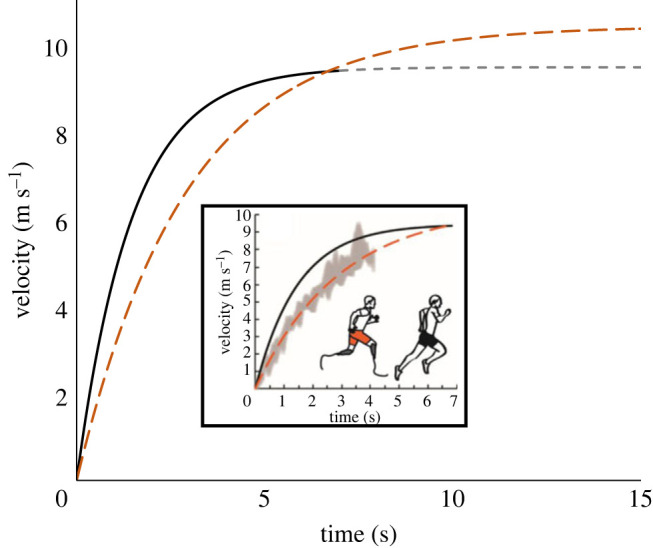
The inset image is a reproduced from Figure 1 (Panel C) in Beck *et al*. showing modelled velocity with respect to time for the NA group (black line) and The Fastest BA (orange line) with the *x*-axis finishing at 7 s. The larger graph depicts our analysis of the velocity curves showing the modelled velocity for NA (black line) and The Fastest BA (orange line) with the *x*-axis extended up to 15 s. NA sprinters achieved greater than 98% of top speed at approximately 6.6 s whereas The Fastest BA did not achieve 98% of his top speed until 13 s. Note that the authors based The Fastest BA's model on a top speed of 10.46 m s^−1^ [2]. However, The Fastest BA's actual top speed is 11.4 m s^−1^ [4], nearly 1.0 m s^−1^ (10%) faster, so the estimate of 13 s to reach top speed is very conservative.

Figure 1 presents two plots: a small inset plot which is reproduced from figure 1 (panel C) in the RSOS publication [4]; and a larger background plot presenting our new analysis. In each plot the *y*-axis is velocity (m s^−1^) and the *x*-axis is time (s). In both plots the black line is the modelled velocity of sub-elite NA sprinters, published in 2005 [5]. The orange line is the modelled velocity of the experimental data from The Fastest BA [4].

The modelled velocity curve for the sub-elite NA sprinters was based on two parameters—mean top speed (9.46 m.s^−1^) and a time constant (1.47 s) [5]. Importantly, these parameters were both obtained from the same maximal 30 m trials and the flattening of the modelled NA velocity curve between 6 and 7 s indicates participants were close (greater than 98%) to their experimentally derived mean top speed of 9.46 m.s^−1^ [4,5]. Other research has found the duration of the maximum acceleration phase in elite sprinters is comparable [6].

Compared with the method used to model maximum acceleration in sub-elite NA sprinters, the method used to model The Fastest BA's acceleration (also figure 1) differed in two essential ways. First, data were acquired from 20 m (rather than 30 m) trials, reducing the distance to accelerate. Secondly, his maximum velocity was not based on measurements obtained from the acceleration trial but estimated using a curve fitting technique. Note that the estimate obtained (10.46 m s^−1^) was nearly 1.0 m s^−1^ slower than Fastest BA's known top speed of 11.4 m s^−1^ [2].

These methodological differences were compounded because, in the RSOS article, the authors only present The Fastest BA's velocity curve up to the point where he reaches the same absolute velocity as the NA sprinters (6–7 s). At this point, the NA sprinters were at 98% of their measured maximum velocity while The Fastest BA was at just over 80% of his estimated maximum velocity. The background plot in figure 1 addresses this shortcoming, extrapolating The Fastest BA's velocity up to his estimated maximum velocity of 10.46 m s^−1^ and capturing his entire acceleration phase [2,4]. The plot indicates that he reaches 98% of his estimated maximum velocity at approximately 13 s, more than 100 m from his start point. Note that these estimates of the time (13 s) and distance (greater than 100 m) for Fastest BA to complete his acceleration phase are conservative because Fastest BA's actual top speed is 11.46 m s^−1^ [2].

When Fastest BA's full acceleration phase is compared with historic results from NA athletes we note the following:

— the duration of The Fastest BA's maximum acceleration phase is at least 13 s, approximately twice the duration that elite [6] and sub-elite [5] sprinters can accelerate maximally; [6]
— compared with elite sprinters, the mean time that it took The Fastest BA to sprint 20 m from a stationary start was 40% slower than the mean for elite sprinters [4,7]. This is 59.5s.d. below the mean for NA sprinters [4,7], more than 17 times the criterion for an outlier, which is ±3.4 s.d. in a sample of ≥10 [8].

In addition to these striking differences, we also note that in figure 2*b* the authors [4] make the point that, at 10 m sec^−1^ Fastest BA had contact lengths that were 7% longer than NA authors [4]. This difference is not trivial. Furthermore, we also note that at 11.46 m/s—Fastest BA's true top speed—visual inspection of the plot indicates a much greater difference between contact lengths for Fastest BA and NA sprinters—approximately 0.10–0.15 m. This is important because it is well-established that greater contact length increases forward running speed [9].

## 3. Concluding remarks

In conclusion, although Fastest BA has run 400 m in times comparable to elite NA 400 m runners, there are striking kinematic differences between NA runners and Fastest BA: his time to 20 m is 59.5 s.d. greater than the mean for NA runners; the duration of his maximum acceleration is approximately 100% greater than NA sprinters; and his contact lengths at top speed are also greater by approximately 0.10–0.15 m. These differences have physiological consequences, particularly in the context of fundamental differences in lower limb anatomy (featuring reduced muscle mass and absent muscle groups and joints) and the use of prosthetics of unregulated materials and design.

We posit that these differences are so large and fundamental that, that for the purposes of athletic competition, running with prosthetic legs and running with biological legs should be considered two different activities and that, for the sake of the integrity of athletics competition, the status quo should be retained (i.e. maintaining separate competitive events for running with and without prosthetics, each with specific, appropriate rules and equipment regulations).

The logic underpinning our position is similar to the logic underpinning the argument for keeping 400 m competitions for wheelchair racers separate from competitions for runners with or without prosthetics: even though elite male wheelchair racers complete 400 m in approximately the same time (43–45 s) as elite runners, the two groups of athletes are performing fundamentally different activities and therefore should not compete together.

## Data Availability

This article has no additional data.
